# Insights from quantitative and mathematical modelling on the proposed 2030 goal for gambiense human African trypanosomiasis (gHAT)

**DOI:** 10.12688/gatesopenres.13070.2

**Published:** 2020-04-21

**Authors:** NTD Modelling Consortium Discussion Group on Gambiense Human African Trypanosomiasis

**Keywords:** gambiense human African trypanosomiasis (gHAT), sleeping sickness, WHO goals, elimination of transmission, NTD Modelling Consortium, prediction

## Abstract

Gambiense human African trypanosomiasis (gHAT) is a parasitic, vector-borne neglected tropical disease that has historically affected populations across West and Central Africa and can result in death if untreated. Following from the success of recent intervention programmes against gHAT, the World Health Organization (WHO) has defined a 2030 goal of global elimination of transmission (EOT). The key proposed indicator to measure achievement of the goal is zero reported cases. Results of previous mathematical modelling and quantitative analyses are brought together to explore both the implications of the proposed indicator and the feasibility of achieving the WHO goal.

Whilst the indicator of zero case reporting is clear and measurable, it is an imperfect proxy for EOT and could arise either before or after EOT is achieved. Lagging reporting of infection and imperfect diagnostic specificity could result in case reporting after EOT, whereas the converse could be true due to underreporting, lack of coverage, and cryptic human and animal reservoirs. At the village-scale, the WHO recommendation of continuing active screening until there are three years of zero cases yields a high probability of local EOT, but extrapolating this result to larger spatial scales is complex.

Predictive modelling of gHAT has consistently found that EOT by 2030 is unlikely across key endemic regions if current medical-only strategies are not bolstered by improved coverage, reduced time to detection and/or complementary vector control.  Unfortunately, projected costs for strategies expected to meet EOT are high in the short term and strategies that are cost-effective in reducing burden are unlikely to result in EOT by 2030. Future modelling work should aim to provide predictions while taking into account uncertainties in stochastic dynamics and infection reservoirs, as well as assessment of multiple spatial scales, reactive strategies, and measurable proxies of EOT.

## Disclaimer

The views expressed in this article are those of the authors. The opinions expressed herein are those of the authors and do not necessarily reflect the views of the World Health Organization. Publication in Gates Open Research does not imply endorsement by the Gates Foundation.

## Background

Gambiense human African trypanosomiasis (gHAT, sleeping sickness) is an infection caused by the parasite *Trypanosoma brucei gambiense*, spread through blood-meal feeding by tsetse in West and Central Africa. Disease symptoms caused by gHAT generally progress over multiple years. Stage 1 disease is defined as the time before the parasite crosses the blood-brain barrier and establishes there, with symptoms such as headache and fever, whereas stage 2 involves neurological symptoms and typically death if left untreated. Fortunately, there are a variety of tools available to assist in the control of gHAT and these have been effective at reducing the burden of gHAT from 37,385 reported cases in 1998 to 953 cases in 2018 ^1^. The primary intervention against gHAT is large-scale, test-confirm-and-treat strategies with diagnosis and confirmation performed by mobile teams in at-risk villages, followed by hospitalisation for treatment; 56% of detected cases in 2016 were diagnosed in this way ^2^. The rest of cases are identified through passive surveillance (self-presentation) in fixed health facilities. There are additional options to reduce transmission by targeting the tsetse vector directly, which has been implemented successfully in several regions, but is currently a non-standard component of intervention strategies in most areas.

Previously, the World Health Organization (WHO) roadmap set a target of elimination as a public health problem (EPHP) for gHAT by 2020 ^2^, which has been redefined as (a) having fewer than 2000 globally reported cases and (b) at least a 90% reduction in areas reporting >1 case per 10,000 people over a five-year period (2016–2020) compared to a 2000–2004 baseline ( Table 1) ^3^. It is complex to determine yet whether the second indicator will be met without a detailed analysis of global data; however, 1419 cases were reported in 2017 and 953 were reported in 2018, suggesting that the first indicator should have been not only met but surpassed. The subsequent WHO goal is global elimination of transmission (EOT) by 2030 ( Table 1) ^4^. Achievement of EOT for gHAT would not only represent a huge stand-alone accomplishment but would place gHAT amongst the very select group of infections for which, through deliberate intervention, global EOT has already been achieved (smallpox and rinderpest) or may be met by 2030 (e.g. Guinea worm and polio).

**Table 1.** Summary of modelling perspectives of the WHO goals for gambiense human African trypanosomiasis (gHAT).

Current WHO Goal (2020 Goal)	Elimination as a public health problem (EPHP). Indicators: (a) <2000 cases globally; and (b) >90% reduction in areas reporting >1 case/10,000 people in 2016–2020 compared to 2000–2004.
Proposed WHO Goal (2030 Goal)	Elimination of transmission (EOT). Indicators: (a) zero reported cases; (b) 90% reduction in high and moderate risk areas relative to 2020 baseline; and (c) >50% and >95% of at-risk populations <1 hour and <5 hours from a health facility with gHAT diagnostics, respectively.
Is the new target technically feasible under the current disease strategy?	The target may be technically feasible using existing tools but perhaps not under the current strategy. EOT may require a step change in the level of surveillance and the use of additional controls (such as door-to-door screening or vector control) in persistent regions. Continued use of existing rapid diagnostic tests, together with 2030 health facility targets, will help case detection. New drugs should improve compliance and ease of treatment.
If not, what is required to achieve the target?	New rapid diagnostic tests, together with 2030 health facility targets, will help case detection. New drugs should improve compliance and ease of treatment. Novel targeted surveillance approaches may be needed close to elimination.
Are current tools able to reliably measure the target?	Existing diagnostics may be sufficient, based on currently reported diagnostic characteristics. However, (i) the indicator of zero reported cases does not imply that the goal of EOT has been reached, (ii) sensitivity could change based on future variation of circulating parasites, and (iii) new tools could improve throughput for large-scale, high-specificity surveillance and/or the ability to detect cryptic human or animal reservoirs.
What are the biggest unknowns?	Prevalence of infection in regions that have never had active surveillance. The role of asymptomatic infections and animal reservoirs as elimination is approached.
What are the biggest risks?	Lack of participation in surveillance at a range of scales. Inability to screen and treat due to conflict. Reduction in controls, particularly passive surveillance, once zero cases are reported locally.

Predictive, mechanistic modelling is a data-driven approach to explore the feasibility of reaching the WHO goals, taking into account the known biology of infection but also representing uncertainty in all processes. Recent mathematical modelling by the NTD Modelling Consortium and collaborators - including groups from the Institute for Disease Modeling, the Swiss Tropical and Public Health Institute, University of Warwick and Yale University - has provided quantitative perspectives on the challenges of reaching and the likelihood of achieving both the 2020 and 2030 WHO goals for gHAT. The models used have been largely deterministic, which typically comprise of systems of ordinary differential equations (ODEs) and describe average expected infection dynamics, however there has recently been implementation of stochastic models, using Gillespie-based simulation algorithms to simulate the impact of chance events as we approach EOT. Stochastic model results will be identified as such, so other cited modelling studies will use deterministic frameworks. The following sections outline some of the key model findings that are of direct relevance to the 2030 EOT goal.

## Modelling insights from strategies previously conducted

In the last two decades, the predominant strategy against gHAT was medical only, comprising active screening and passive surveillance followed by treatment. Current medical-based gHAT control strategies are working well in reducing incidence ^2^ and modelling indicates they are also reducing underlying transmission ^5, 6^. Shortening time to detection and treatment of cases further reduces morbidity and subsequent onward transmission ^7^. Modelling indicates that, in Uganda and South Sudan, passive surveillance reduced transmission by 30–50% during the 1990s and 2000s; strengthening these systems in gHAT endemic regions could therefore have great potential ^8^. Staged gHAT case data (differentiating between stage 1 and stage 2 cases) can provide substantial information on the effectiveness of, and changes in, the passive surveillance system. Usually the proportion of stage 1 cases is low in passive surveillance (~30% in 2012 ^9^); due to the lack of symptom severity and specificity in stage 1, and thereby limiting the self-presentation of those infected and passive diagnoses made for people in this stage. Conversely, most active detections (mass screening) are in stage 1 (~70% in 2012 ^9^) as case confirmation relies on serology and parasitology, rather than symptoms. Improvement in time to detection in former Bandundu province in the Democratic Republic of Congo (DRC) is reflected in a greater proportion of stage 1 cases, with modelling estimating a possible doubling of the stage 1 passive detection rate between 2000–2012 ^10^.

Despite these successes, controls can be disrupted by conflict or other events; notably, the Ebola epidemic in West Africa resulted in temporary cessation of medial activities ^11^. Furthermore, in higher endemicity settings or regions with little screening, the current medical-only interventions are predicted to be insufficient for achieving EOT by 2030 (e.g. in several health zones in Bandundu, DRC, EOT is predicted to be realised after 2050) ^12– 14^. In these settings - assuming scale up of vector control (VC) is feasible and the substantial (>80%) reduction in tsetse density ^15, 16^ can be reproduced widely - supplementing medical interventions with VC is predicted to be cost-effective at relatively low willingness-to-pay (WTP) thresholds in high-risk areas ^14^, and to lead to EOT in much shorter timescales (1–6 years instead of >30 years in some settings) ^12, 13^. It is noted that deterministic modelling studies are unable to exactly predict when transmission will be eliminated and therefore models have employed a proxy threshold of <1 new infection per 100,000 or 1,000,000 per year. Whilst this proxy is imperfect, more recent stochastic modelling indicates that stochastic and deterministic model dynamics for gHAT follow very similar trends even at low prevalence ^17^. Furthermore, whilst deterministic modelling may also be unsuitable for some small-scale modelling, stochastic modelling of gHAT in villages finds a population size of around 2,000 is sufficient for persistence, whereas this “critical community size” for persistence of other infections is typically much higher, e.g. around 300,000 people for measles ^18^; this indicates that deterministic gHAT models at the health zone level (100,000 people) pose limited cause for concern.

## What are the practical implications of the elimination of transmission goal?

The WHO 2030 goal for gHAT is EOT globally, with the key proposed indicator of achieving zero reported cases ( Table 1). Other proposed indicators relate to sustaining coverage of passive surveillance.

### Measuring the target

In the long term, reaching EOT will lead to zero detected cases; however, the two objectives are not equivalent - it is possible either that zero case detections could occur without EOT or, conversely, that gHAT detections could be observed even after EOT.

***EOT before zero reporting.*** Achieving EOT may not immediately lead to zero detected cases as there is often a long period between infection and detection (several years is typical ^19^, although in extreme cases this could be decades ^20^). As EOT is approached, the choice of confirmatory diagnostics becomes increasingly important as imperfect test specificity, even current, multidiagnostic algorithms with ~99.9% specificity (serological testing followed by microscopy), can lead to false positive cases. As we approach the 2030 goal, more rigorous methodologies (e.g. the laboratory-based trypanolysis test with 100% specificity ^21^) should help to circumnavigate this problem of positive predictive value.

**Figure 1.**
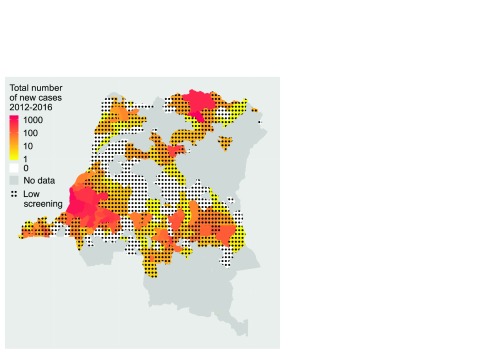
Geographic availability of gambiense human African trypanosomiases (gHAT) data across the Democratic Republic of the Congo. Colours represent numbers of reported cases in health zones from the last five years of data set. Health zones that never (2000–2016) report cases or active screening are coloured grey, whilst zones with <5% mean active screening coverage (during 2012–2016) are shown with black dots. This figure has been adapted from data presented in Franco *et al*. ^1^ under a CC-BY 4.0 license and with permission from Dr Erick Mwamba Miaka, director of the National HAT Control Programme (PNLTHA) of the Democratic Republic of the Congo.

***Zero reporting but not EOT.*** Achieving zero detected cases does not mean that there is EOT for numerous reasons. The first possibility is that screening does not identify all remaining infections at peri-elimination. Only some of the population at risk is regularly screened; modelling ^5, 6, 10^ suggests that some high-risk individuals (~20% of the population) may not attend active screenings and data show that not all settlements in high-risk areas are screened annually (around 50% of villages in a high-endemicity region of DRC were screened in any given year ^4, 18^). Coverage may improve with mini mobile teams (screening otherwise inaccessible villages) or door-to-door screening (likely increasing the number of high-risk people participating), but pockets of infection could still be missed. Furthermore, large areas of DRC ( Figure 1), South Sudan and Central African Republic with potential transmission are not regularly screened due to regional conflicts. Secondly, even where there is a functional health system, there is a high probability of underreporting; models for Bandundu, DRC, suggest that only around 20% of gHAT cases that escape active detection are identified by passive detection (Model W in Castaño *et al*. ^10^), corresponding to ~63% of all infections being unreported. Choice and used of available diagnostics are crucial for information certainty – as we approach the endgame it may be that current diagnostics become less able to detect circulating antibodies (due to changing parasite antigen expression) and therefore decreased sensitivity of surveillance tools.

**Figure 2.**
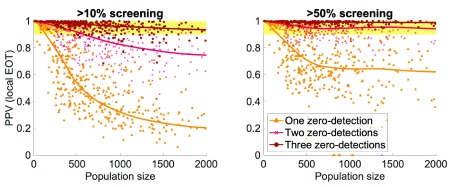
Probability of elimination of transmission (EOT) at the village-level based on case reporting. The positive predictive value (PPV) of zero case detections to assess whether local EOT has occurred is the probability of zero human infection given consecutive active screenings with no cases found and no passive reporting in between (for >2 screenings). Model parameterization is for Yasa Bonga and Mosango health zones in the Democratic Republic of the Congo. The left figure uses all active screenings with >10% coverage, while the right figure is restricted to screenings of >50% coverage. The yellow region indicates >90% confidence that EOT has been met locally. This figure has been reproduced with permission from Davis *et al*. ^18^.

Stochastic models have been used in order to explore the predictive power of one or more years of zero detected cases in estimating the likelihood of EOT at the health zone level (~100,000 people) for the DRC, finding that three years of zero reported cases is sufficient to have >80% positive predictive value (PPV) that EOT has been met ^17^. Another study with stochastic village-level model (simulating observed active screening from 2000–16 for 559 settlements in Yasa Bonga & Mosango, Bandundu, DRC) also examined detecting zero cases under active screening. This smaller-scale model strongly indicates that three or more consecutive rounds of finding zero cases is sufficient to reach >90% PPV of local EOT across typical village sizes and where screenings that achieve <10% coverage were ignored ( Figure 2) ^18^. There is higher certainty of EOT in smaller settlements and only using active screenings with >50% coverage as a measure could reduce the number of screening rounds needed to have high confidence. Current WHO guidelines recommend conducting three consecutive years of active screening with zero detections in a village before stopping ^7^, therefore providing high confidence of local EOT prior to cessation. Modelling suggests that factoring screening coverage and population size into future guidelines could further improve certainty that EOT is met before stopping and could reduce the number of zero detections required for smaller settlements if coverage is sufficient. Scaling these insights between different spatial scales is confounded by spatial correlations and reinvasion, suggesting an intelligent and reactive surveillance methodology is required.

Finally, there is potential for circulation of infection in animal reservoirs or persistence in asymptomatic individuals, which could lead to resurgence even after zero human reporting ^22^ and is discussed in more detail in the “Risks and unknowns” section below.

### Technical feasibility

Models predict that for some regions (e.g. Equateur, DRC) continuation of the current medical-only strategy could achieve local EOT by 2030 ^12^; however, in other regions (particularly some of Bandundu, DRC) this strategy may need to be supplemented with additional or improved interventions, even in areas likely to meet EPHP by 2020 ^13^. Some drivers of local epidemiology that explain these different outcomes include variation in tsetse density and heterogeneity of risk of exposure within regions, in addition to the diversity in implementation and coverage of active screening. Local EOT may be unsustainable without continued control due to the risk of reinvasion from other infected areas.

Multiple modelling approaches have shown that improvements to passive surveillance, targeting active screening to include high-risk groups, and VC could all result in reduced transmission and lead to EOT by 2030 with higher probability than the current medical-only strategy ^10, 23^. Whilst it may not be necessary to implement VC across all settings, modelling consistently finds that VC averts infections fastest amongst the considered strategies. Modelling also suggests that the use of other new technologies (i.e. new oral drugs and RDTs) could lead to EOT but with lower probability and slower timelines than VC ^14^.

### Operational feasibility

Modelling results are generally based on assumptions that the health system retains similar or better functionality over the next 10 years. However, political instability, conflict, or a reduced priority for tackling the reduced number of future cases could all lead to less control being applied in the future. Modelling can also simulate the possible impact of such future disruption to activities (planned or otherwise) in addition to more optimistic assumptions about intervention coverage. Whilst planned cessation following zero reporting has already been considered in some modelling studies ^17, 18^, unplanned intervention suspensions and its impact can and should be explored in future modelling work.

### Ability to sustain achievement of the goal

Stopping large-scale control activities against gHAT too soon could be problematic for EOT. Modelling was used to explore potential resurgence following attainment of EPHP in Guinea ^24^, concluding that the presence of animal reservoirs would likely lead to resurgence following cessation of screening and vector control, but resurgence was unlikely if transmission was anthroponotic (see “Animal reservoirs” below). Indeed, interruption of medical interventions in Guinea during the Ebola outbreak has shown that early cessation of activities in low prevalence settings can still lead to resurgence over three years ^11^. In contrast, regions that maintained VC but stopped medical intervention observed a decrease in prevalence during the same time period ^25^.

Even if an area has reached local EOT, there is a concern that cessation of activities could be risky if nearby places have on-going transmission. Stochastic modelling of reinvasion of gHAT in villages in DRC that have achieved local EOT suggests short-term reinvasion is likely (>70%) from a single infected person, but less likely to cause persistent infection for ~15 years (<20%) ^18^. This is due to the high probability that someone will be passively detected and treated or die before creating secondary human infections (through tsetse) even in the absence of active screening. This is also reflected in basic reproduction numbers which only slightly exceed one.

### Considerations of costs and allocative efficiency

The burden of NTDs falls in resource-poor settings, and it is of utmost importance to efficiently use the resources available. Combining cost models with dynamic transmission models provides a valuable framework in which to examine the financial and economic impacts and the cost effectiveness of strategies which account for changing burden as elimination is approached and/or achieved. One such cost-effectiveness analysis for gHAT across settings of different transmission intensities has found that VC combined with other new technologies (diagnostics and drugs) is likely to be highly cost-effective in high-transmission settings (i.e. cost-effective for WTP thresholds >$386/disability-adjusted life year [DALY] averted). In moderate-transmission settings this strategy is only likely to be cost-effective for high WTP thresholds (>$1509/DALY averted), with medical-only strategies using new technologies likely to be preferable for lower WTP thresholds ^14^. Unfortunately, cost-effectiveness in this traditional net-benefits framework does not always align with the goal of EOT by 2030, as strategies that are cost-effective (in terms of DALYs versus costs) may not be sufficient to meet the EOT goal. For example, in Sutherland *et al*. ^14^, VC strategies were generally required to have high predicted probability of EOT by 2030, despite having low probabilities of being cost-effective in moderate- or low-transmission settings.

An analysis on the affordability of gHAT intervention and patient financial impact estimated that the total costs of a global control or elimination programme would be substantial (depending on the programme, between US$410.9 million and US$1.2 billion, compared to US$630.6 million for control activities in 2013–2020) 2 ^6^. Alleviation of impoverishment and catastrophic health expenditures for households due to gHAT infection can only be achieved through elimination, rather than control, programmes. Ongoing work by the co-authors as part of the HAT Modelling and Economic Predictions for Policy (HAT MEPP) project seeks to assess the cost-effectiveness of elimination strategies based on recent, local data and model updates in order to provide specific and up-to-date recommendations across different settings. It is anticipated that, as before, recommended strategies will not be the same in different transmission settings or geographic regions and will depend on affordability and willingness to pay for averted DALYs or EOT.

## Risks and unknowns faced by gHAT elimination programmes

A more complete review of key factors that may impact the EOT goal is given by Büscher *et al*. ^22^. Here, insights arising from modelling-based studies are discussed.

### Systematic non-participation in screening

Several modelling studies suggest that there is systematic non-participation in active screening, with high-risk individuals less likely to participate; the models without this heterogeneity were unable to match the observed longitudinal patterns of cases across different regions ^5, 6^. More detailed data on age and gender of screening participants and gHAT cases could help better elucidate key groups in the population most responsible for transmission.

### Animal reservoirs

Although prevalence of gHAT infection in animals is nonzero, estimates are uncertain and the role of infected animals in onward transmission is unclear ^22^. One modelling study matched to point prevalence data from animals in Cameroon suggested that animals constitute a possible transmission reservoir, implying that control targeting only human cases would be unable to eliminate gHAT due to persistence in animals ^27^, whereas modelling using longitudinal human data (Guinea, DRC, Chad) suggest that there is comparable statistical support for models with and without an animal reservoir ^5, 6, 24^. However, animals are unlikely to be able to sustain transmission on their own in Chad ^5^. The model used for Cameroon used the next generation matrix approach and the assumption of constant endemic levels of infection, in conjunction with sampled animal prevalences. In contrast the other models only utilised human case data, and fitted to decreasing reporting trends and time-varying intervention activities. Table 2 highlights some future work and data which would be needed to provide greater certainty on animal reservoirs, although it is noted that different geographies may have different potential based on human-tsetse-animal abundance and contact patterns.

**Table 2.** Immediate priorities for modelling for gambiense human African trypanosomiasis (gHAT).

Priority issue / question identified by WHO during this meeting	How can modelling address this?
**Probability of interrupted transmission:** Can existing mathematical models be used to define the probability of interruption of gHAT transmission in regions where no cases have been detected?	Using historic data, and assumptions on current passive surveillance, models can be generated that capture the observed dynamics at regional foci and calculate the probability (positive predictive value, PPV) of interrupted transmission given that no cases have been reported for different periods of time.
**Reactive screening:** How does a reactive screening strategy compare to active screening and passive detection, or passive detection alone in terms of: - reduction of transmission and associated timescales? - case reporting?	Modelers can develop/refine modelling of current active and passive strategies to simulate a reactive screening strategy. - The spatial scale considered will impact results. - Reactive strategies can and should be included in cost predictions and cost- effectiveness analyses
**Animal reservoir:** - What do we know about their role in transmitting disease? - How could an animal reservoir affect the 2030 target?	Some modelling has already explored possible animal reservoirs. Modelers can continue to explore: - Whether there are signals of animal reservoirs by assessing human case data alone - If there is any support for these models, to assess the relative contribution of animals to transmission, and what impact this could have on timescales to achieve EOT - To include animals in a village-scale model framework (to assess PPV of zero case detections in active screening on EOT) - To make estimates more robust by fitting to human and animal data (if available) - To assess implications of animal reservoirs in decision analyses between interventions
**Asymptomatics:** - Can we estimate the potential number of asymptomatic infections? E.g. for one detected case, how many remain undetected? - How likely are asymptomatics to infect others? - What do we know about their role in (maintaining) transmission?	- Existing modelling frameworks can be adapted to include potential asymptomatics (including self-cure or skin infections) - Sensitivity analysis and/or matching to data (if available) could estimate possible numbers of asymptomatics, their relative contribution to transmission, infection timescales, and relative infectivity. Lack of data may lead to large confidence intervals - Modellers can evaluate the effectiveness of different strategy types in models with and without asymptomatic people - e.g. would we select the same intervention strategy if asymptomatics play a substantial role in transmission?
**Spatial prediction:** Support defining areas that should be screened, where there is potential of transmission. Similarly, can we rule out certain areas?	- A tsetse absence model could be used to assess regions which are unlikely to have gHAT due to unsuitable habitat. - This can be used to explore the joint distribution of the active and passive surveillance data and to look for factors/variables which could predict the underlying variation and probability of reporting. - It may be possible to include a range of factors into these predictions including changing population distribution and land-use.

The observed decreasing human case trends combined with model fitting to such data provide optimism that there is limited (if any) transmission from non-human animals to humans (through tsetse); however the discovery of transmission cycles in dogs in the last phase of the Guinea worm eradication programme ^28^ serves as an important reminder that the role of animals should not yet be completely discounted as we aim towards EOT for gHAT. Modelling suggests that VC, including spraying livestock, would reduce any possible transmission from animals, although pockets of sustained transmission could occur away from human activities 2 ^9^.

### Asymptomatic reservoirs

Asymptomatic infections in humans have been considered in a few transmission models. In some ^10, 23^, the role of these infections in maintaining transmission or causing resurgence was not directly assessed, although one modelling study using data from Guinea found both asymptomatic and clinical human infections were necessary for gHAT to persist (assuming no animal reservoir) and concluded that passive surveillance alone was not sufficient for gHAT monitoring in the approach to elimination ^30^. Generally there is little routinely collected data to help inform asymptomatic transmission modelling. Some case data will contain asymptomatics – while all parasitological-confirmed infections are reported, symptoms are not recorded in aggregated data – but screening diagnostics may be less sensitive on such infections.

### Movement

So far, little attention has been paid to the movement of people in the modelling literature on gHAT; however, this may be important in areas that recently achieved disease-free status. Particular regions of concern would include formerally endemic areas with both high influxes of refugees/internally displaced people and limited surveillance.

## Immediate priorities

Table 2 highlights a list of priority questions for modellers that are of relevance for the 2030 EOT goal for gHAT arising from discussions between modellers and WHO. In the table the questions are identified from a policy perspective by WHO, and the modellers provide the model and data which would be required to address the policy questions using modelling. Similar tables are provided for other NTDs as part of this special collection.

## Data availability

No data are associated with this article.
