# Supplementary material for: Insights from quantitative and mathematical modelling on the proposed 2030 goal for gambiense human African trypanosomiasis (gHAT)
Source: Gates Open Res. 2020 Apr 21;3:1553. Originally published 2019 Oct 7. [Version 2] doi: 10.12688/gatesopenres.13070.2 (PMC7193711; doi:10.12688/gatesopenres.13070.2)
Supplement: Peer review history

# Peer review: Reviewer response for version 2

**DOI:** 10.21956/gatesopenres.14304.r28781

**Published:** 2020-04-23

**Authors:** Mario Recker

**Affiliations:** 1 College of Engineering, Mathematics and Physical Sciences, University of Exeter, Penryn, UK

I have read through the revised article and think the authors have done a great job responding to my original comments and improving the manuscript accordingly. I'm happy with the current version to be accepted for indexing.

Does the article adequately reference differing views and opinions?

Yes

Are all factual statements correct, and are statements and arguments made adequately supported by citations?

Yes

Is the Open Letter written in accessible language?

Yes

Where applicable, are recommendations and next steps explained clearly for others to follow?

Yes

Is the rationale for the Open Letter provided in sufficient detail?

Yes

Reviewer Expertise:

NA

I confirm that I have read this submission and believe that I have an appropriate level of expertise to confirm that it is of an acceptable scientific standard.

# Peer review: Reviewer response for version 1

**DOI:** 10.21956/gatesopenres.14204.r27989

**Published:** 2019-11-07

**Authors:** Mario Recker

**Affiliations:** 1 College of Engineering, Mathematics and Physical Sciences, University of Exeter, Penryn, UK

This open letter provides an overview of the use of mathematical modelling of gHAT and its uses in support of the WHO’s target of global elimination of transmission by 2030. Coming from a modelling background but with little knowledge of gHAT I read this letter with much interest. However, what I feel is currently lacking is a bit more detail and structure regarding the models and their predictions themselves. That is, from the article it is clear that various models have been developed and that they have been applied to answer slightly different gHAT-related questions. What is not clear is what types of models have been used for what questions, where do models generally agree or disagree, what are crucial knowledge gaps highlighted by the these exercises, and what is the direction that models should be heading to support local and global elimination efforts. I believe that much of this could be achieved simply by re-organisation/restructuring, making the sections more focused and less overlapping. For example, the problems of a potential animal reservoirs or asymptomatic infections come up more than once, the same with screening/diagnostics. Personally I would focus on just a few key obstacles, important aspects of gHAT epidemiology/elimination, or intervention measures, detailing the problem and then illustrating what modelling has found and/or how it can be used in the future. Also, the authors mention in the Abstract that accounting for uncertainties and stochastic effects is very important, which I fully agree with, especially as one approaches disease elimination scenarios. However, I was missing the discussion on this in the text; are none of the approaches developed so far stochastic/deal with uncertainty? Given its importance I would suggest that the authors could maybe dedicating a separate (sub-)section on this?

 More specific comments:

- Background, second paragraph: maybe this is a bit negative but saying that 963 cases suggests that the first indication has been greatly surpassed the goal of getting it below 2000 is a bit of an exaggeration; to me this suggests things are on target
- Modelling insights from [...], first paragraph: it is not immediately obvious how staged gHAT case data can provide substantial information on the effectiveness of surveillance; more detail on this would be welcome
- Table 1: the question ‘is the new target technical feasible [...]’ is answered by ‘yes’; but the next row goes into ‘if not, what is required’ - given that the first answer is yes, do we need this then? Or is the answer not really ‘yes’
- EOT before zero reporting: what are ‘algorithms’ with 99.9% specificity?
- Zero reporting but not EOT: this seems to me like a two-part problem, the first is general under-reporting, and the other long periods of asymptomatic infections; would it be worth making this distinction?
- Zero reporting but not EOT, second paragraph: replace ‘replaying’ with ‘simulating’
- Technical feasibility: explain why the strategy could achieve local EOT in some regions but not in others
- Ability to sustain achievement [...]: it seems like the issue about the potential animal reservoir is slightly controversial, i.e. what’s the evidence for or against?
- Ability to sustain achievement [...], last paragraph: why is reintroduction less likely to cause persistent infections? Presumably control programs would stop after elimination has been achieved?
- Asymptomatic reservoirs: this is a clear example where it would be good to have more of an overall discussion on where models agree, where they differ and why.
- Table 2: do you need the second column, or would it be possible to have this table just listing/detailing (top priority) questions that can be addressed/answered with modelling? Personally I find that the ‘how’ is not that important, especially as no other model details and assumptions are being discussed here 

Overall I think that this is an important piece of work highlighting the uses of mathematical models in public health, and a little more structured and focused approach would make it even better.

Does the article adequately reference differing views and opinions?

Yes

Are all factual statements correct, and are statements and arguments made adequately supported by citations?

Yes

Is the Open Letter written in accessible language?

Yes

Where applicable, are recommendations and next steps explained clearly for others to follow?

Yes

Is the rationale for the Open Letter provided in sufficient detail?

Yes

Reviewer Expertise:

NA

I confirm that I have read this submission and believe that I have an appropriate level of expertise to confirm that it is of an acceptable scientific standard, however I have significant reservations, as outlined above.

# Response

**Published:** 2020-04-02

**Authors:** Kat Rock

Thank you for your helpful comments, we have added several clarifications to the manuscript as suggested (see below), and although we do not consider that complete article restructuring is necessary we have included more cross-referencing to link different sections of the article. 

- To improve our clarity about model types we have firstly added the following text to the background section “The models used have been largely deterministic, which typically comprise of systems of ordinary differential equations (ODEs) and describe average expected infection dynamics, however there has recently been implementation of stochastic models, using Gillespie-based simulation algorithms to simulate the impact of chance events as we approach EOT.”

We also note the proxy thresholds required if assessing predictions of EOT in deterministic frameworks in our “Modelling insights” section: “It is noted that deterministic modelling studies are unable to exactly predict when transmission will be eliminated and therefore models have employed a proxy threshold of <1 new infection per 100,000 or 1,000,000 per year. Whilst this proxy is imperfect, more recent stochastic modelling indicates that stochastic and deterministic model dynamics for gHAT follow very similar trends even at low prevalence (18). Furthermore, whilst deterministic modelling may also be unsuitable for some small-scale modelling, stochastic modelling of gHAT in villages finds a population size of around 2000 is sufficient for persistence, whereas this “critical community size” for persistence of other infections is typically much higher e.g. around 300,000 people for measles (19); this indicates that deterministic gHAT models at the health zone level (100,000 people) pose limited cause for concern.”

- To clarify the importance of staged case data we have added: “Usually the proportion of stage 1 cases is low in passive surveillance (~30% in 2012 (10)); due to the lack of symptom severity and specificity in stage 1, and thereby limiting the self-presentation of those infected and passive diagnoses made for people in this stage. Conversely, most active detections (mass screening) are in stage 1 (~70% in 2012 (11)) as case confirmation relies on serology and parasitology, rather than symptoms. Improvement in time to detection in former Bandundu province in the Democratic Republic of the Congo (DRC) is reflected in a greater proportion of stage 1 cases, with modelling estimating a possible doubling of the stage 1 passive detection rate between 2000-2012 (9).”
- In Table 1 we have moved some of the technical feasibility text into the first box, although our uncertainty about strategies rather than tools themselves means we have left creation of novel screening strategies in the “if not” box. 
- The simulations for reintroduction include cessation of screening activities, however very low R_0 values (just above 1) mean that local extinction is likely from a single case. We added: “This is due to the high probability that someone will be passively detected and treated or die before creating secondary human infections (through tsetse) even in the absence of active screening; this is also reflected in basic reproduction numbers which only slightly exceed one.”
- For asymptomatic reservoirs we change “unclear” to “not directly assessed” to highlight that the models don’t necessarily disagree, but this has not generally been explored. We also add an additional sentence to explain why the issue of asymptomatics has been studied infrequently.
- We would like to retain the second column in Table 2 for two reasons. Firstly, the questions themselves were highlighted by WHO as priorities, irrespective of whether modelling and data are at a suitable stage to answer them. Our perspective on the data needs and modelling requirements to answer them indicate how readily modelling can be used to address these questions. Secondly, this letter is part of a special collection in which a similar table is provided for each NTD. We have added additional text in the immediate priorities section to explain the table more clearly.

# Peer review: Reviewer response for version 1

**DOI:** 10.21956/gatesopenres.14204.r27987

**Published:** 2019-10-29

**Authors:** Michael P. Barrett

**Affiliations:** 1 Wellcome Centre for Integrative Parasitology, Institute of Infection, Immunity and Inflammation, University of Glasgow, Glasgow, UK

The open letter is a clear overview of modelling efforts tracing the road to elimination of gambiense HAT.

- Background: Paragraph 1, line 6:  note parasites can cross the BBB before establishing themselves there to make stage 2 infection, so please change to reflect that.
- Line 11: After 37,385 - add the word "reported" before cases.
- Paragraph 2, line 4:  2,000 (add the comma). Line 9:  Also add a comma to 1,419.
- Paragraph 3, line 10-11: place commas around "and likelihood of reaching".
- Table 1, point 5: I am not certain that "existing diagnostics are likely sufficient". If skin parasites are not sustaining serum antibody titres, and also if the standard antigens used in current tests are not present in a cohort of residual parasites it is likely the diagnostics will become ever less sensitive.
- On page 4: the reference to the trypanolysis test which purports 100% specificity is, I think, equally vulnerable to loss of sensitivity in cases where parasites not expressing antigens central to this test are in circulation.
- On page 5: Second paragraph points to the potential of animal reservoirs and asymptomatic individuals. However, the downward trend appears to have been following predictions quite nicely (where these refugia are not considered). Does the modelling so far, therefore, rule out a significant impact of the animal reservoir and asymptomatic patients, or does incidence have to get even lower before the problem will become manifest?
- Page 5. section Operational feasibility: It is stated that "Models are therefore making assumptions that screening and other controls follow recent trends." Can't the models themselves be used now to predict what happens in the event of different removal of control scenarios?
- Page 6, Last paragraph before the "Risks and unknowns....." section: This covers estimate of cost of control based on the Sutherland and Tediosi work. Clearly these estimates could have a large impact on policy decisions. Can the authors offer an opinion on how robust they consider those findings (in the light of modelling predictions?)
- Page 6, column 2, section on "Movement", line 4:  "formally" should be "formerly"
- References, reference 1: More information needed to access.
- Some references e.g.  4, 12,14, 15, 20, 21, 26 are using capital first letters for individual words in the title, while others are not.

Does the article adequately reference differing views and opinions?

Yes

Are all factual statements correct, and are statements and arguments made adequately supported by citations?

Yes

Is the Open Letter written in accessible language?

Yes

Where applicable, are recommendations and next steps explained clearly for others to follow?

Yes

Is the rationale for the Open Letter provided in sufficient detail?

Yes

Reviewer Expertise:

African trypanosomiasis, drugs mode of action and resistance mechanisms

I confirm that I have read this submission and believe that I have an appropriate level of expertise to confirm that it is of an acceptable scientific standard.

# Response

**Published:** 2020-04-02

**Authors:** Kat Rock

Thank you for your helpful comments, we have added additional clarifications to the manuscript as recommended:

- We agree we should be more careful with our wording in Table 1 about diagnostics. We have updated the text to: “Existing diagnostics may be sufficient, based on currently reported diagnostic characteristics. However, (i) the indicator of zero reported cases does not imply that the goal of EOT has been reached,  (ii) sensitivity could change based on future variation of circulating parasites, and (iii) new tools could improve throughput for large-scale, high-specificity surveillance and/or the ability to detect cryptic human or animal reservoirs. “
- To address the possibility for decreasing sensitivity of diagnostics we added in the “Zero reporting but not EOT” section: “Choice and use of available diagnostics are crucial for information certainly – as we approach the endgame it may be that current diagnostics become less able to detect circulating antibodies (due to changing parasite antigen expression) and therefore decrease sensitivity of surveillance tools.”
- We discuss animal reservoirs primarily on page 6 (whilst mentioning them on page 5) so have now added a cross-reference “and is discussed in more detail in the “Risks and Unknowns” section below.” In that section we add a note on decreasing case trends increasing our optimism: “The observed decreasing human case trends combined with model fitting to such data provide optimism that there is limited, if any, transmission from non-human animals to humans (via tsetse), however the discovery of transmission cycles in dogs in the last phase of Guinea worm eradication programme (26) serves as an important reminder that the role of animals should not yet be completely discounted as we aim towards EOT for gHAT.“
- You are correct that models can be used to predict cessation or removal of controls and we have now added the following: “Modelling can also simulate the possible impact of such future disruption to activities (planned or otherwise) in addition to more optimistic assumptions about intervention coverage. Whilst planned cessation following zero reporting has been already considered in some modelling studies (Davis, 2019), unplanned intervention suspension and its impact can and should be explored in future modelling work “
- Firstly, we would like to emphasise that the cited cost-effectiveness study did utilise both a cost and transmission dynamic modeling framework, therefore it accounted for a decreasing burden as predicted by the transmission model for different interventions. (We have added a sentence to clarify this - “Combining cost models with dynamic transmission models provides a valuable framework in which to examine the financial and economic impacts and the cost-effectiveness of strategies which account for changing burden as elimination is approached and/or achieved. One such cost-effectiveness analysis…”) We also added some text on our ongoing work which highlights important analyses for providing updated and localised strategy recommendations although we believe the overall message will hold: “Ongoing work by the co-authors as part of the HAT Modelling and Economic Predictions for Policy (HAT MEPP) project seeks to assess the cost-effectiveness of elimination strategies based on recent, local data and model updates in order to provide specific and up-to-date recommendations across different settings. It is anticipated that, as before, recommended strategies will not be the same in different transmission settings or geographic regions and will depend on affordability and willingness to pay for averted DALYs or EOT.”
